# Spontaneous bilateral intraorbital hematoma: A particular form of sickle cell disease complications in children

**DOI:** 10.1002/ccr3.5994

**Published:** 2022-06-24

**Authors:** Haritanjona Andriamiarintsoa, Herveat Ramanandafy, Orlando Andoniaina Andriamiadanalisoa, Koloina Randriantianarisoa, Prosper Harinarivo Randrianarivelo, Emmylou Prisca Gabrielle Andrianah, Lova Hasina Ny Ony Narindra Rajaonarison, Léa Raobela, Joëlson Lovaniaina Rakotoson, Hanta Marie Danielle Vololontiana, Ahmad Ahmad

**Affiliations:** ^1^ Department of Medical Imaging and Radiodiagnosis University Hospital of Joseph Ravoahangy Andrianavalona Antananarivo Madagascar; ^2^ Department of Internal Medicine University Hospital of Joseph Raseta Befelatanana Antananarivo Madagascar; ^3^ Department of Ophthalmology University Hospital of Joseph Ravoahangy Andrianavalona Antananarivo Madagascar

**Keywords:** hematoma, hemoglobinopathy, Madagascar, orbital cellulitis, visual acuity

## Abstract

Spontaneous bilateral intraorbital hematoma is a rare complication of sickle cell disease in children. Imaging examinations are of paramount importance in the diagnosis and conditioning of the management processes in order to avoid complications that can compromise the visual function prognosis.

## Graphical abstract

Spontaneous orbital hematoma can be the initial presentation in patients with bleeding abnormalities and other blood dyscrasias. The visual prognosis is at stake in the absence of immediate treatment. The management mostly involves observation and only in certain cases may require surgical intervention.

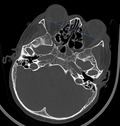

## 1 INTRODUCTION

Sickle cell disease is an inherited hemoglobinopathy characterized by vaso‐occlusive crises. Intraorbital hematoma secondary to hematopoietic medullary infarction is a rare complication of sickle cell disease.^1^ The visual prognosis is at stake in the absence of immediate treatment. We report an atypical form of intraorbital hematoma revealed by bilateral exophthalmos in a sickle cell child; then, we will highlight the role of imaging in the diagnosis and management of intraorbital hematoma.

## 2 CASE REPORT

**FIGURE 1:**
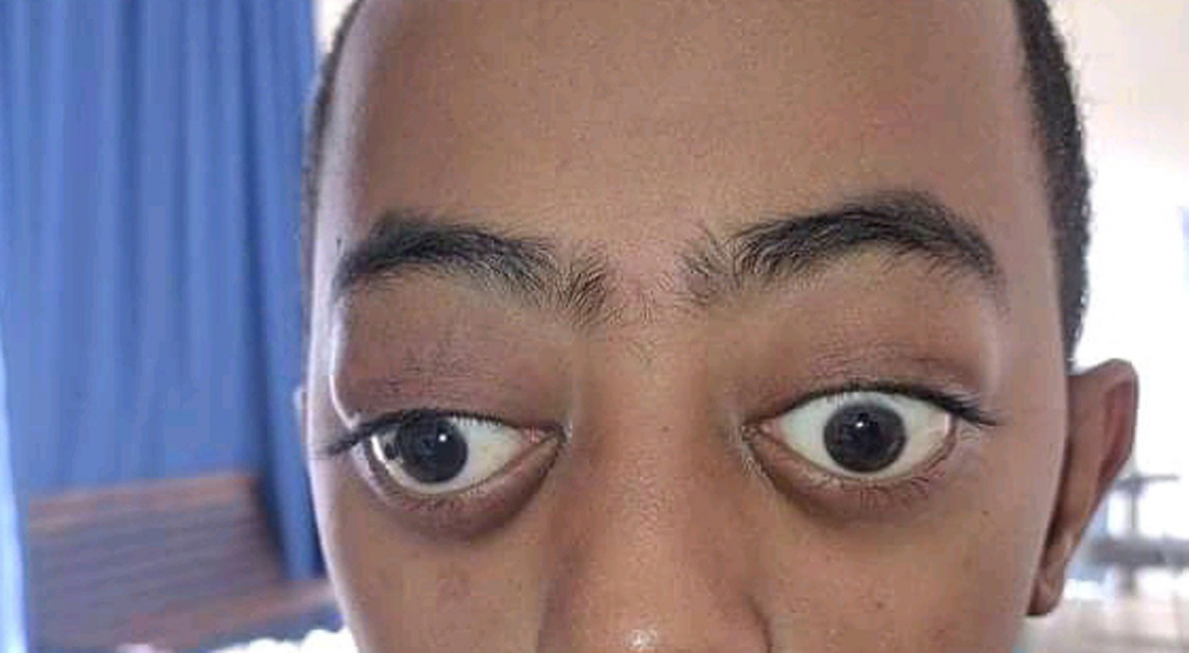
Exophthalmos with diplopia and bilateral palpebral edema

**FIGURE 2:**
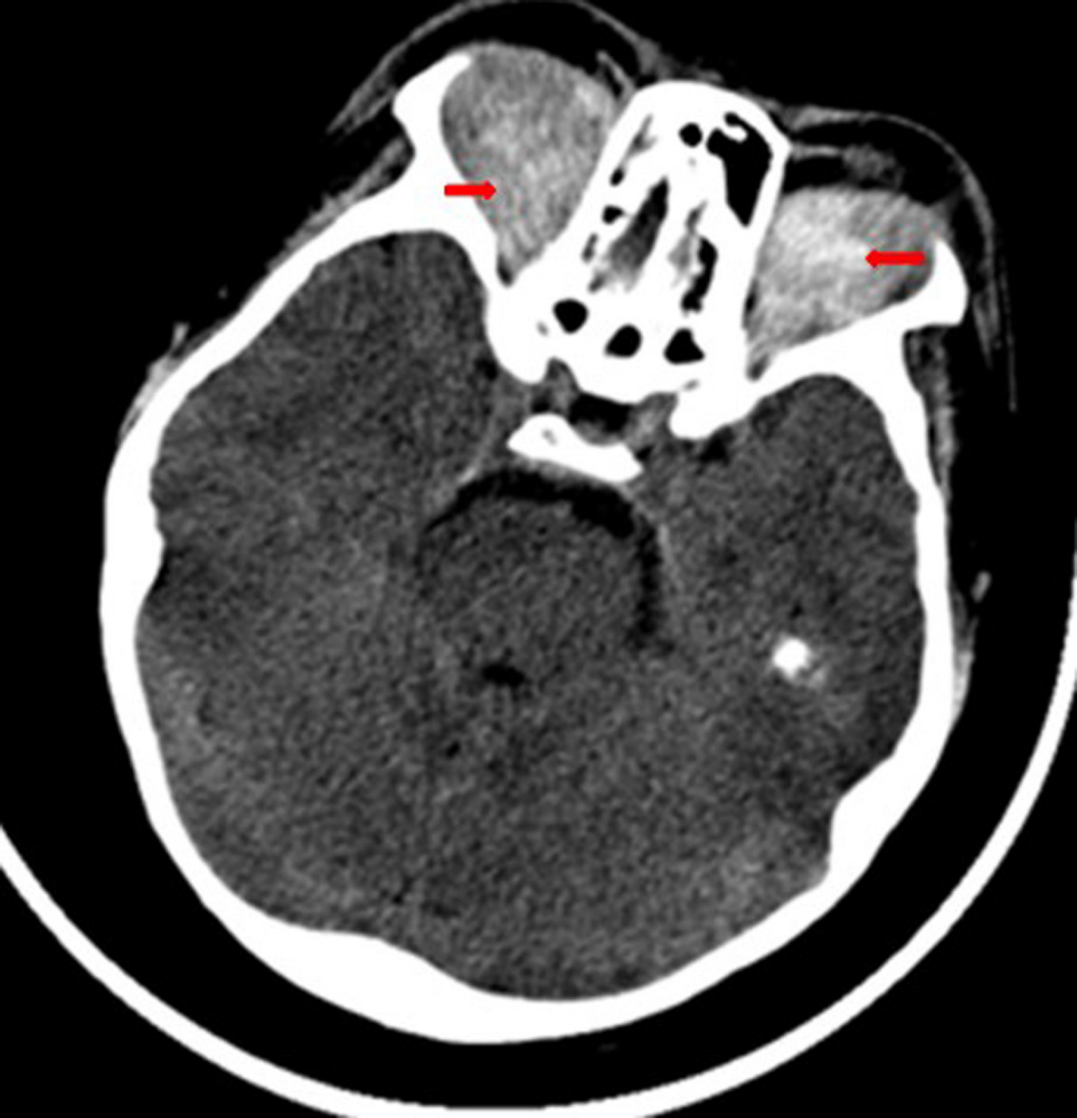
Oculocerebral CT scan with parenchymal window: bilateral intraorbital formation that is spontaneously hyperdense, consisting of both intra‐ and extraconal compartments, and of hematic density

**FIGURE 3:**
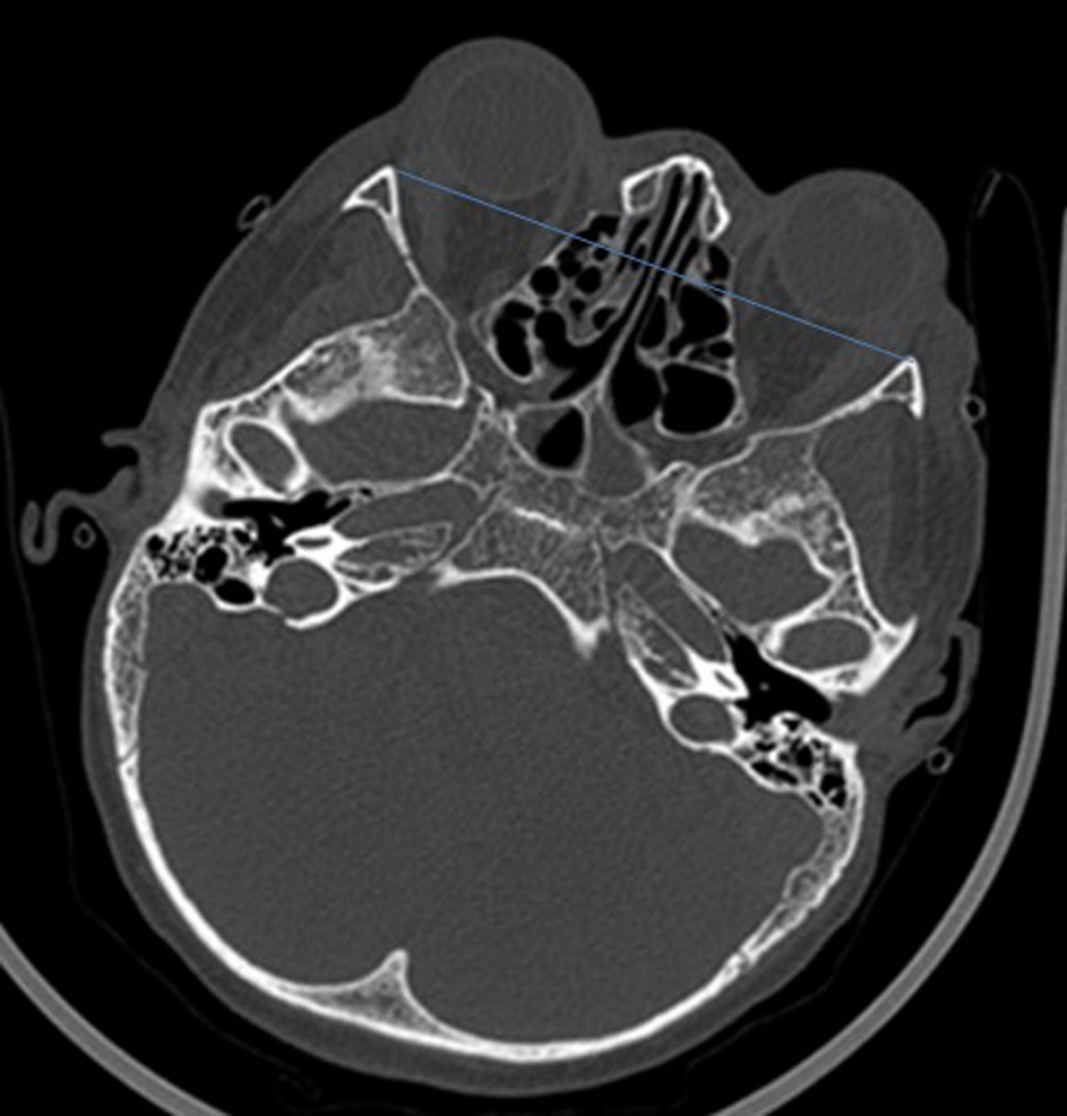
Oculocerebral CT scan with bone window: exophthalmia stage 3, with the absence of abnormalities in bone structure

**FIGURE 4:**
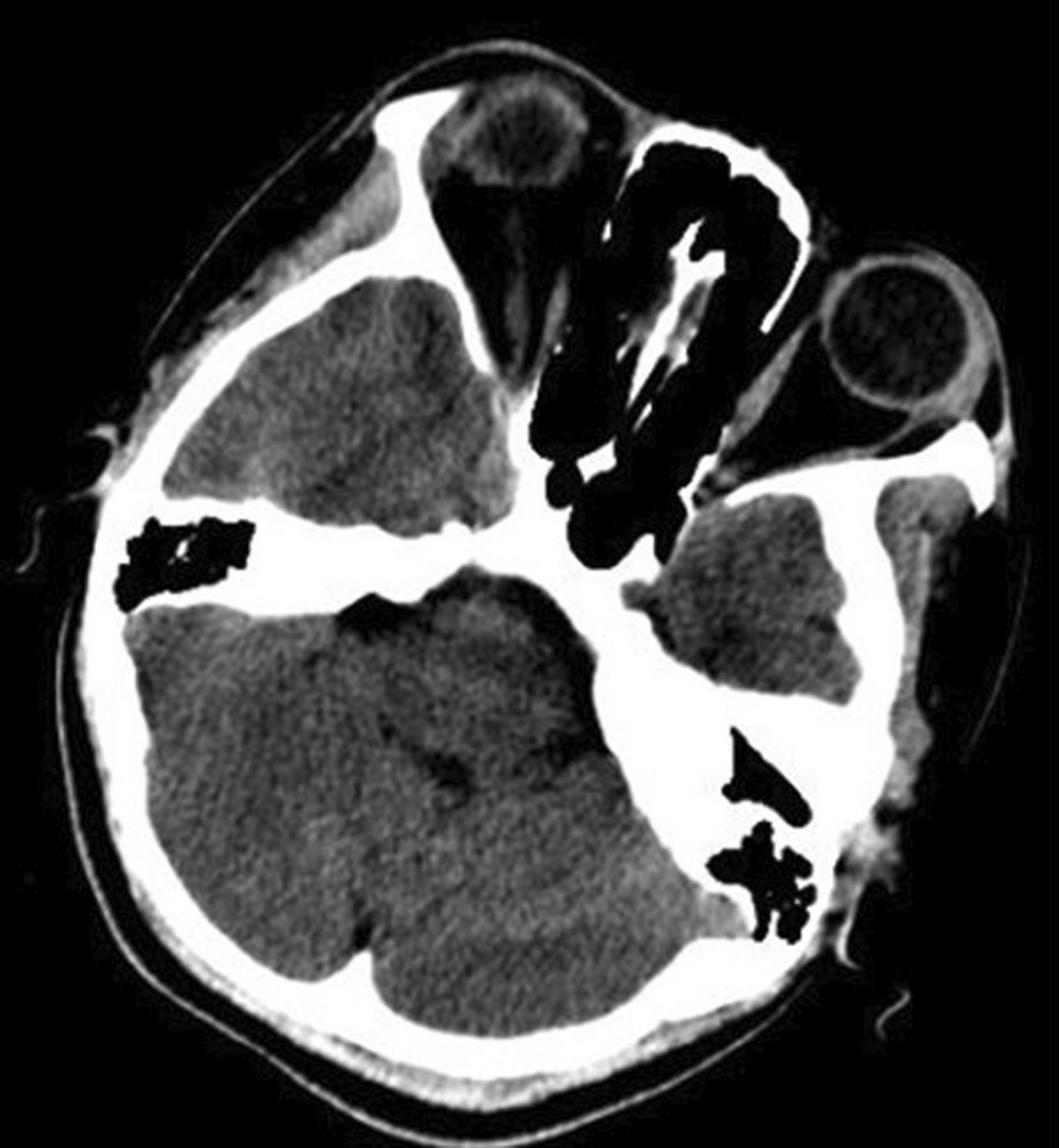
Oculocerebral CT scan with parenchymal window: regression of the intraorbital hematoma

**FIGURE 5:**
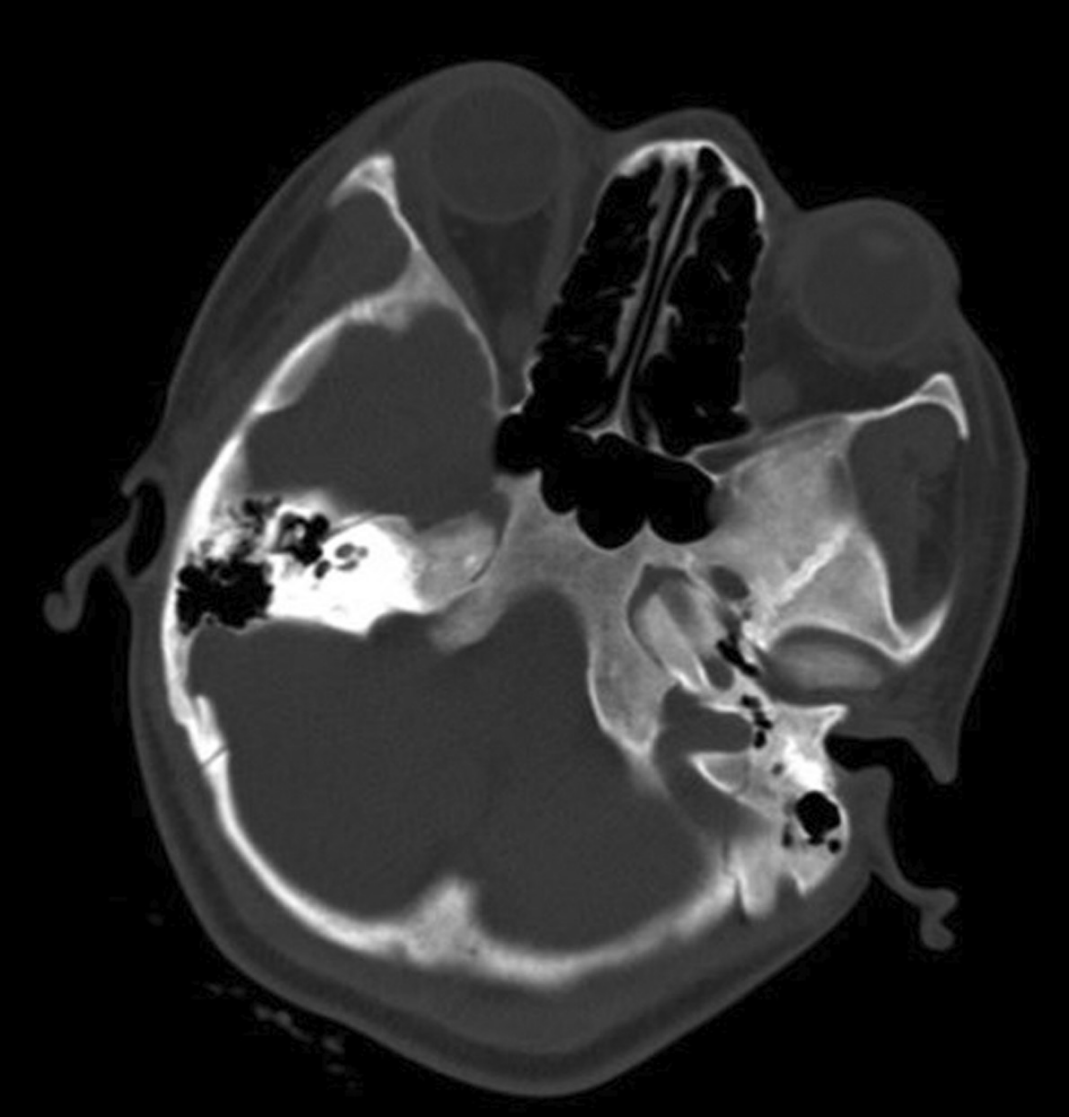
Oculocerebral CT scan with bone window: total disappearance of exophthalmos

A 15‐year‐old boy, known to have homozygous sickle cell disease, came for a consultation with headache and bilateral eye pain, which had been evolving for 2 weeks in a context of apyrexia. In his background, there was no notion of craniofacial traumatism or recent surgery. On ophthalmological examination, an ocular motility limitation in certain gazes, a palpebral edema, and a bilateral painful exophthalmos that is non‐pulsatile and non‐reducible were noted (Figure 1). The measured visual acuity was altered to 5/10 P3 for the right eye and 7/10 P2 for the left eye. The fundus examination was very limited and did not show any particularities. Other examinations, especially neurological, were unremarkable. Blood cell count (BCC) showed a hemoglobin (Hb) level at 9.3 g/dl, a neutrophil polynuclear (NP) level at 6.5 × 10^9^/L, and a platelet (Plt) level at 377 × 10^9^/L. The C‐reactive protein (CRP) level was discretely elevated at 7 mg/L. The coagulation profile (prothrombin time (PT): 15.4 s, prothrombin level (PL): 95%) was normal. Oculocerebral CT with parenchymal window showed a bilateral intraorbital structure spontaneously hyperdense, density hematic, in intra‐ and extraconjunctival situation, and pushes forward and downward the oculary globes (Figure 2). In the bone window, an oculocerebral CT scan showed a bilateral exophthalmos at grade 3 without abnormal bone structure (Figure 3). Ocular B‐mode ultrasound was performed but was very limited because of the narrowing of the acoustic window due to the exophthalmos. In front of the anamnestic, clinical, and scanographic context, the diagnosis of a spontaneous bilateral intraorbital hematoma in a sickle cell patient was evoked. The patient was put on oral corticosteroids for 5 days. And in front of the ocular pain, a treatment containing analgesic was instituted. The evolution was favorable after 1 week, marked by the regression of the exophthalmos and a progressive recuperation of visual acuity and oculomotricity. The oculocerebral CT scan at 1 week showed regression of the intraorbital hematoma (Figure 4). The recuperation of visual acuity and oculomotricity ad integrum was achieved after 1 month with the total disappearance of the exophthalmos (Figure 5). The patient was then readmitted to the sickle cell management center.

## 3 DISCUSSION

In the orbit, the hematopoietic medullary space is smaller. It is therefore rare to find orbital bone infarcts in sickle cell patients.^1^ About thirty cases are described in the literature with an average age of 14 years, half of which had an intraorbital hematoma.^2^ The singleness of our case in Madagascar would therefore allow comparison with the literature and could provide additional data on the diagnosis and management of intraorbital hematoma in children with sickle cell disease. The bilateral form is rarely reported in the literature. In a series conducted by Sokol, the bilateral form represents one third of reported cases.^2^ This one makes our case a particular form of intraorbital hematoma. Exophthalmos is a reflection of the mass effect; it is the main mode of discovery of the intraorbital hematoma. The first functional manifestations are ocular pain and localized edema. In the absence of urgent management, the visual prognosis is at risk in case of optic nerve compression.^1^, ^3^ In front of the rapid installation of the exophthalmia, especially if it is bilateral, the differential diagnosis is vast. Among these, we can cite orbital pseudotumor, orbital rhabdomyosarcoma, optic nerve glioma, orbital extension of bilateral retinoblastoma, and bilateral orbital cellulitis. In all these possibilities, imaging studies are of paramount importance in characterizing the nature of the compressive mass. In our case, the hematic density was spontaneously hyperdense on the CT scan, suggesting the diagnosis of a bilateral intraorbital hematoma. The magnetic resonance imaging, which would determine the hematic nature of the collection,^4^ is not accessible; therefore, the CT scan remains an examination that retains its place, although it is relatively non‐specific. However, the absence of sinus pathology and the location of the collection on the lateral wall of the orbit are the arguments against an abscess^2^ except that the clinical and biological examinations are sterile. Medical treatment seems to be sufficient in most cases, including treatment of the crisis and the use of corticosteroids by some teams with caution in the context of sickle cell disease.^1^, ^2^ Surgery drainage is reserved for cases of persistent optic nerve compression threatening the visual prognosis.^2^, ^5^ In our case, the treatment is purely medical, making the exophthalmos disappear after 1 month with the recovery of the visual acuity ad integrum.

## 4 CONCLUSION

Spontaneous bilateral intraorbital hematoma secondary to bone infarction is a rare but serious complication of sickle cell disease. Imaging examinations hold an important place in the diagnosis and have an implication in the management of preserving the visual function. A regular follow‐up of sickle cell disease with the implementation of a background treatment and the precaution of factors favoring vaso‐occlusive attacks could avoid this complication, which sometimes has a poor prognosis.

## AUTHOR CONTRIBUTION

HA, OAR, and KR followed up the patient and collected the clinical data. HR collected the clinical data and drafted the report. PHR followed up the patient. EPGA and LHNONR designed and critically revised the report. LR, JLR, HMDV, and AA validated the report. All the authors have read and approved the final draft of the manuscript, contributed to this work, and approved the final version.

## CONFLICTS OF INTERESTS

None declared.

## ETHICAL APPROVAL

The article does not contain any personal information that could identify the patient. The names and dates on the chest CT scan have been hidden. The authors have included only information necessary for scientific understanding.

## CONSENT

Written consent was obtained from the patient for publication of this case report and accompanying images.

## Data Availability

Data are available on request from the corresponding author.
